# BEAN 2.0: an integrated web resource for the identification and functional analysis of type III secreted effectors

**DOI:** 10.1093/database/bav064

**Published:** 2015-06-27

**Authors:** Xiaobao Dong, Xiaotian Lu, Ziding Zhang

**Affiliations:** State Key Laboratory of Agrobiotechnology, College of Biological Sciences, China Agricultural University, Beijing 100193, China

## Abstract

Gram-negative pathogenic bacteria inject type III secreted effectors (T3SEs) into host cells to sabotage their immune signaling networks. Because T3SEs constitute a meeting-point of pathogen virulence and host defense, they are of keen interest to host–pathogen interaction research community. To accelerate the identification and functional understanding of T3SEs, we present BEAN 2.0 as an integrated web resource to predict, analyse and store T3SEs. BEAN 2.0 includes three major components. First, it provides an accurate T3SE predictor based on a hybrid approach. Using independent testing data, we show that BEAN 2.0 achieves a sensitivity of 86.05% and a specificity of 100%. Second, it integrates a set of online sequence analysis tools. Users can further perform functional analysis of putative T3SEs in a seamless way, such as subcellular location prediction, functional domain scan and disorder region annotation. Third, it compiles a database covering 1215 experimentally verified T3SEs and constructs two T3SE-related networks that can be used to explore the relationships among T3SEs. Taken together, by presenting a one-stop T3SE bioinformatics resource, we hope BEAN 2.0 can promote comprehensive understanding of the function and evolution of T3SEs.

**Database URL:** http://systbio.cau.edu.cn/bean/

## Introduction

T3SEs are proteins secreted by Gram-negative pathogenic bacteria to interfere with host immune signaling networks (1, 2). They are secreted into host cells through type-III secretion systems (T3SSs) (1), which are encoded by animal and plant pathogenic bacteria, such as *Salmonella typhi*, *Escherichia coli* O157:H7, *Yersina enterocolitica*, *Pseudomonas syringae* pv *tomato* DC3000 and *Ralstonia solanacearum*. By blocking the immune signaling pathways at specific subcellular locations, these cytotoxic proteins are thought to assist pathogenic bacteria in evading the attacks from immune systems (3, 4). T3SEs have evolved diverse functional domains (5) to mimic the functions of host cell proteins or covalently modify them (2, 4). However, the biochemical mechanism used by T3SEs can be very different from their counterparts in host cells. For example, *Shigella* T3SE OspF can irreversibly remove phosphate group from phosphothreonine residue in mitogen-activated protein kinases Erk1/2 or p38 by converting threonine into dehydrobutyrine (6). This strategy has not been found in enzymes from eukaryotic cells. Moreover, the evolution of T3SEs seems also unusual. A previous study (7) showed that pathogenic bacteria can generate new T3SEs through terminal reassortment of existing T3SE sequences, implying complex evolutionary relationships among T3SEs. Recently, it has been realized that protein intrinsic disorder regions, flexible segments without fixed 3D structure, are evolutionary hallmarks of T3SEs (8).

The unique functions of T3SEs make them not only the powerful weapons of pathogens but also useful probes for researchers to investigate mechanisms of host immunity. Systematical characterization of the repertoires of T3SEs in pathogenic bacteria is helpful to identify the main virulence strategy commonly adopted by different pathogenic bacteria as well as the evolutionary relationships among different T3SEs (9–11). With the rapid development of high-throughput sequencing technologies, more and more genomes of pathogenic bacteria have been fully sequenced (11). There is an unprecedented requirement for bioinformatics tools/resources that can accurately identify and conveniently analyze T3SEs from these genomic data. To this end, a few state-of-the-art bioinformatics methods have been developed to predict T3SEs (12–19). Meanwhile, there also exist several excellent T3SE databases (e.g. T3SEdb (20), Effective (21) and T3DB (22)), although they are mainly designed to store/predict T3SE sequences and provide limited analysis tools to further annotate T3SEs. Moreover, the relationships among different T3SEs are hardly explored by them. With the accumulation of more T3SE data, we anticipate the development of more comprehensive T3SE web resources is still highly required.

We previously developed a machine-learning predictor BEAN (Bacterial Effector ANalyzer) to identify T3SEs from pathogen genomes. In this predictor, the compositions of evolutionarily conserved amino acid (AA) pairs (23) were used to represent N-terminal secretion signals in T3SEs (17). Since BEAN was released in 2013, its web server has predicted >35 000 protein sequences submitted by users from ∼30 countries. Despite BEAN having shown good performance, there is still room for improvement. Indeed, some useful information was overlooked in the original version of BEAN. First, traditional sequence alignment-based search usually gives a reliable prediction if the query protein is very similar to a known T3SE. Second, the unique functional domains harboring on T3SEs can also be useful to discriminate T3SEs and non-T3SEs. Third, although the type III secretion signal (1) is believed to reside within the N-terminal of T3SEs in most cases, C-terminal is also required for the secretion of some T3SEs. For example, the C-terminal region (residues from 321 to 409) of *Salmonella* T3SE SipC is essential for its translocation into HeLa cells (24). The six residues (519–524) of C-terminal is required for efficient secretion of T3SE Tir in *E. coli* (EHEC O157:H7) (25). Other cases include *Salmonella* T3SEs SifA (26) and SipB (27).

**Figure 1.**
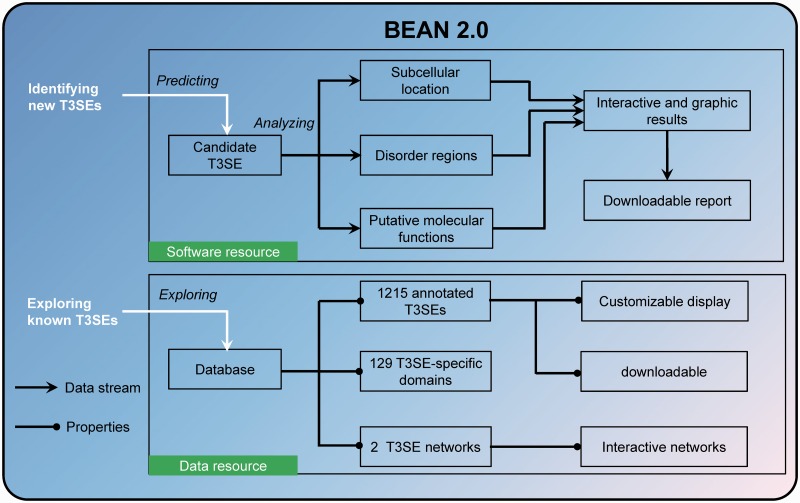
Overview of the resources in BEAN 2.0.

Here, we developed BEAN 2.0 as an integrative web resource of T3SEs (Figure 1). In addition to integrating the above information to improve the accuracy of T3SE prediction, BEAN 2.0 also provided multiple functional analysis tools to assist users in annotating putative T3SEs conveniently. Moreover, BEAN 2.0 compiled 1215 verified T3SEs from 221 pathogenic bacteria into a database and it also provided two networks that can be interactively visualized to explore the relationships among different T3SEs. Through providing a one-stop bioinformatics service, we hope BEAN 2.0 can accelerate the identification and analysis of new T3SEs.

## Materials and Methods

### Data collection

We collected 1202 T3SEs from the Uniprot database (version of 2014.01). CD-hit (http://weizhong-lab.ucsd.edu/cd-hit/) with the sequence identity cutoff of 40% was used to remove similar sequences. As a result, 249 T3SEs were retained. Among them, six T3SEs have sequence lengths <100, which were further skipped. To obtain a 1:2 ratio of positive to negative samples, 486 negative samples were randomly selected from the non-T3SE dataset used in our previous work (17), which was compiled from eight well-studied Gram-negative bacterial proteomes by several criteria. The pairwise sequence identity among negative samples was also controlled as ≤40% using CD-hit. Finally, we obtained 243 T3SEs and 486 non-T3SEs, which constitute the major benchmark dataset in this work. The partition of the benchmark dataset across 5-fold cross-validation test, the independent test and the genome-wide test, will be detailed in the following sections.

### Overall workflow of T3SE prediction in BEAN 2.0

Using a similar strategy as described by Kumar *et al.* (28) in mitochondrial protein prediction, BEAN 2.0 consists of three components: sequence alignment-based predictor, domain-based predictor and machine-learning predictor. The compiled non-redundant benchmark dataset is used to derive this system.

A query protein sequence will firstly be processed by the sequence alignment-based predictor. It tries to search for the most similar sequence of the query one in the training data using BLAST. If a highly similar sequence is found, the corresponding label (T3SE or non-T3SE) will be assigned to the query protein. If no similar sequence is detected with threshold E-value ≤0.01, the query protein will be switched to the domain-based predictor. The domain-based predictor scans the query protein sequence (E-value ≤1e-5) and compares its Pfam domains with the domains compiled from the training dataset. First, we collected the Pfam domains of the training dataset. Then we divided the domains into three types: (i) exclusively T3SE domains only observed in T3SEs; (ii) exclusively non-T3SE domains only observed in non-T3SEs and (iii) shared domains observed in both of T3SE and non-T3SE. If the query protein harbors T3SE-exclusive domains (non-T3SE-exclusive domains), it will be predicted as a T3SE (non-T3SE); otherwise, it will be further processed by the machine-learning predictor. The parameters used in BLAST and Pfam scan were preliminarily optimized according to the suggestions in Kumar *et al.*’s work (28).

In the machine-learning predictor, the homologs of the query protein are searched firstly through HHblits (29) for constructing a sequence profile. Then, the resulting profile will be divided into three parts: N-terminal 2–51 AAs, 52–121 AAs and C-terminal 50 AAs. Since the first N-terminal AA is methionine in most bacterial protein sequences, it is ignored in this step. A 1600-dimension feature vector is extracted using the profile-based *k*-spaced amino acid pair composition (HH-CKSAAP) (17) to represent each of the three parts. The only exception is when the length of the middle part is <70 AAs, a 1600-dimension zero vector is used to encode this part. Then, the 4800-dimension feature vector is input into a linear Support Vector Machine model to predict the label of the query protein. The linear SVM model was also learned from the training dataset and the corresponding SVM parameters were the same as our previous work (17).

### Evaluation the performance of BEAN 2.0

To assess the performance improvement of the hybrid strategy, we conducted a 5-fold cross-validation test on five models, including BEAN, BEAN*, BEAN*+BLAST, BEAN*+Pfam and BEAN 2.0. Note that BEAN stands for the original BEAN method, BEAN* represents the model trained by 4800-dimension vectors, BEAN*+BLAST means the combination of BEAN* and sequence alignment-based predictor, and BEAN*+Pfam indicates the combination of BEAN* and domain-based predictor. We randomly divided the benchmark dataset into a training dataset containing 200 T3SEs and 400 non-T3SEs and an independent test set containing 43 T3SEs and 86 non-T3SEs. The 5-fold cross-validation test on the training dataset was carried out. We further compared BEAN 2.0 with four existing T3SE predictors through the independent test. We trained a BEAN 2.0 model based on the whole training dataset and used it to predict the independent dataset. The standalone programs of EffectiveT3 (13), BPBAac (16) and T3_MM (18) were downloaded from their websites to predict the independent test dataset. Since the standalone version of ANN (14) is not available, we used the web server directly to do the prediction.

We collected the T3SEs on two independent genome datasets from a plant pathogen *Pseudomonas syringae* pv. *phaseolicola* 1448a (*P. syringae*) (29) and an animal pathogen *E. coli* O157:H7 (*E. coli*) (30). We chose these two pathogens because most of T3SEs in their genomes have been systematically screened. To ensure fair comparison, we removed the T3SEs of the query species from our training set. We collected 23 and 48 known T3SEs in 5045 proteins of *P. syringae* and 5255 proteins of *E.* *coli*. After removing the protein sequences containing < 100 residues, we obtained 22 and 45 known T3SEs in 4626 proteins of *P. syringae* and 4475 proteins of *E.* *coli*. The four existing T3SE predictors were also used to screen T3SEs from these two genomes.

### Performance assessment parameters

In the 5-fold cross-validation test or the independent test, we used five parameters [sensitivity, specificity, accuracy, F1-score and Matthew correlation coefficient] to evaluate the performance. These parameters are defined as:

specificity=TNTN+FPsensitivity=TPTP+FNaccuracy=TP+TNTP+FP+TN+FNF1−score=2×TP2×TP+FP+FNMCC=TP×TN−FN×FP(TP+FN)×(TN+FP)×(TP+FP)×(TN+FN)

where TP, FP, TN and FN stand for the number of true positives, false positives, true negatives and false negatives.

With respect to genome-wide tests, we mainly used the median of the known T3SEs’ ranks to compare different predictors. We also listed the ranks of known T3SEs in the whole genomes of these two species. For BEAN 2.0, we prioritized all positive prediction results in the whole genome as: sequence alignment-based prediction > domain-based prediction > machine-learning prediction. The prediction results from the same method were further sorted according to E-values (sequence alignment-based prediction, and domain-based prediction) or prediction scores (machine-learning prediction). For the other four existing predictors, their output scores were used to rank prediction results.

### Construction of BEAN 2.0 web server

The core algorithm was developed by PERL program and the construction of web server was based on LAMP (Linux+Apache+MySQL+PHP), an open-source software frequently used to build high-availability websites. The prediction model used in the website was trained with the whole benchmark dataset (i.e. all 243 T3SEs and 486 non-T3SEs). The 1202 T3SEs originally collected for constructing the BEAN 2.0 model and the other 13 T3SEs newly collected during the development of the web server were included in our T3SE database.

## Results and Discussion

### Accurate prediction of T3SEs using BEAN 2.0

To test the usefulness of the hybrid approach (Supplementary Figure S1), we compared the performance of our original BEAN and the prediction models integrating with sequence alignment, domain information or the sequence composition beyond N-terminal. We found these three types of information can stably improve the sensitivity of prediction. Further test showed that the performance improvement of the hybrid approach should not be attributed to the similarity of N-terminal sequences in our training data (Supplementary Table S1).Through the 5-fold cross-validation test on the training dataset, BEAN 2.0 achieved a sensitivity of 92.00% and a specificity of 97.00% in comparison to the 80.00% and 96.75% of our original BEAN model (Supplementary Figures S2 and S3). We also demonstrated there is no significant overfitting caused by the high feature dimensionality in BEAN 2.0 (Supplementary Figure S4 and Table S2). Although many existing predictors only use the N-terminal signal sequence, our results indicate that from the practical point of view the other sequence regions can also facilitate the identification of T3SEs.

**Figure 2.**
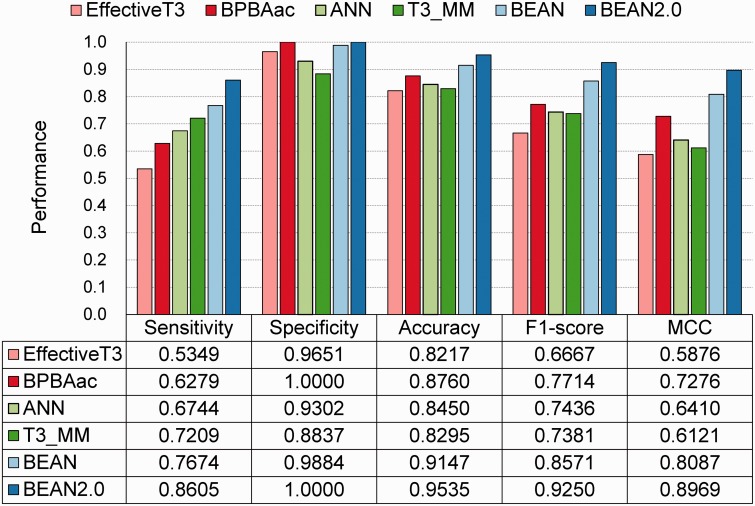
The performance of different T3SE predictors on the independent dataset.

The performance of BEAN 2.0 was compared with five existing methods through the independent dataset (Figure 2). As shown in Figure 2, BEAN 2.0 achieved the best accuracy value of 95.35%, which is 13.18%, 7.75%, 10.85%, 12.40% and 3.88% higher than EffectiveT3 (13), BPBAac (16), ANN (14), T3_MM (18) and BEAN. The good performance of BEAN 2.0 was also confirmed in other five different independent dataset selection scenarios (Supplementary Table S3). Generally, BEAN 2.0 reveals a very stable improvement in specificity and sensitivity.

Moreover, we tested these methods on two independent genome datasets from a plant pathogen *P. syringae* and an animal pathogen *E. coli*. For 22 and 45 known T3SEs in 4626 proteins of *P. syringae* and 4475 proteins of *E. coli* (>100 AAs), BEAN 2.0 successfully predicted 21 and 33 of them in the top 50 ranked candidates according to their scores. The median of known T3SEs’ ranks in BEAN 2.0 results is 18.5 and 35 for *P. syringae* and *E. coli*, respectively. Regarding the other methods, T3_MM gave the second best result with a median rank of 37.5 in *P. syringae*, and BEAN gave the second best result with a median rank of 132 in *E. coli*. The obvious advantages shown in the genome-wide T3SE predictions indicate that BEAN 2.0 has a practical applicability in T3SE screening (Detailed comparison results are provided in Supplementary Tables S4–S6).

The major performance improvement of BEAN2.0, especially for the increased sensitivity, can be ascribed to the following two aspects. First, BEAN2.0 takes the sequence composition of the middle and C-terminal into account with the purpose of incorporating more possible type-III secretion-related information, such as chaperone-binding sites and potential C-terminal secretion signal. The information may increase the chance of detecting more T3SEs. Second, although the overall performance of BEAN is better than BLAST or Pfam, it has also some drawbacks. BEAN tries to model the sequence composition of all the training data and learns the most stable characteristics that can be used to distinguish T3SEs and non-T3SEs. This process strengthens the common distinctive characteristics, but it also weakens the ones that can only be observed in a handful T3SEs. As sequence alignment-based methods, on the contrary, BLAST and Pfam can sensitively detect some unique sequence patterns shared by a few T3SE proteins. Due to the methodological complementary between BEAN and BLAST/Pfam, the integration of them can result in a substantial performance improvement.

### An integrative T3SE web resource

An integrative analysis platform is valuable to further investigate potential functions of bacterial secretion proteins (31). Therefore, in addition to T3SE prediction, BEAN 2.0 also provides other three types bioinformatics resources to facilitate T3SE function research, including a sequence annotation suite, a curated T3SE database and two functional relationship networks constructed from the known T3SEs.

BEAN 2.0 uses sequences in FASTA format to predict T3SE candidates. Users can submit 200 sequences in one job at most. The job name is required and the email address is a voluntary choice. If the email address is provided, BEAN 2.0 will send an email containing the URL of the prediction result when the job is finished. Generally, it takes ∼3 min to conduct T3SE prediction for a query sequence. For genome-wide T3SE prediction, a command-line version can be downloaded and deployed on users’ local machine and run in the multi-threading mode.

The prediction results can be directly transferred to sequence annotation suite for subcellular location prediction, domain annotation or long disorder region detection. The default subcellular location predictor is the widely used TargetP (32). But considering only three different location information (mitochondrion, chloroplast and secretion proteins) can be given in TargetP, we also provide an alternative choice using Cell-PLoc package (33) whose prediction covers up to 22 subcellular locations. For domain annotation and protein disorder region analysis, Pfam (34) and IUPred (35) were used. The users are also allowed to submit new sequences in FASTA format through ‘Analysis’ button. Sequence annotation results will be shown in interactive figures and tables. All of the results are allowed for downloading. Registered users can keep their query sequences confidential and manage their jobs. Although we encourage users to register with BEAN 2.0, the web server also allows anonymous use.

### T3SE databases and T3SE-related networks

**Figure 3.**
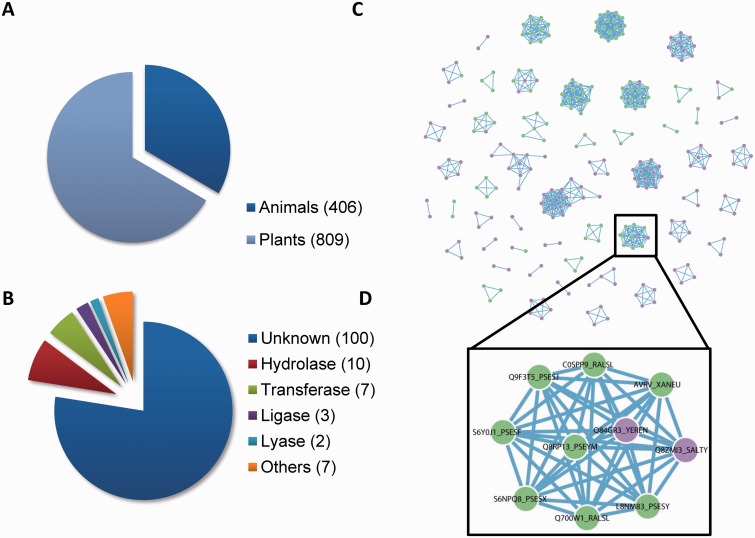
The composition of T3SE database in BEAN 2.0. (**A**) The proportion of T3SEs from animal and plant bacteria. (**B**) The functional categories of T3SE domains. (**C**) The T3SE network (i.e. Effector-Net). Two T3SEs are connected if they share a common Pfam domain. T3SEs from plant bacteria are colored as green, while T3SEs from animal bacteria are colored as purple. (**D**) T3SEs from both plant and animal bacteria are connected through YopJ domain (Pfam ID: PF03421).

Our database includes 1215 curated T3SEs from 221 pathogenic bacteria (Figure 3A and B), displaying T3SE name, source organism, sequence length, experimental status, Pfam domain and subcellular locations in host. Users can search them through an interactive Javascript table. To facilitate the database update, we also encourage users to contribute newly discovered T3SEs to our database through ‘Contribution’ dialog box.

In addition, BEAN 2.0 provides the T3SE-specific domain information extracted from known T3SE. The information is shown in a form, including the exclusively T3SE Pfam domains and its possible function.

Inspired by a previous study that used protein homology networks to visualize the evolution of T3SSs (36), we constructed two interactive networks (Effector-Net and Domain-Net) to visualize the relationships among T3SEs and T3SE domains, respectively (Figure 3C and Supplementary Figures S5 and S6). In Effector-Net, each T3SE is represented as a node and two T3SEs are connected if they share a common domain. The nodes marked in green (purple) represent that they are secreted by plant pathogens (animal pathogens). The current Effector-Net covers 261 non-redundant T3SEs and 832 edges. These 261 T3SEs consist of 52 connected subnetworks. In Domain-Net, each Pfam domain harboring on known T3SEs is represented as a node and two domains are connected if they concurrence on a T3SE. The current Domain-Net included 74 different Pfam domains and 59 edges among them. Most domains do not connect with any other, indicating that they are exclusively observed in only one non-redundant T3SE. The most highly connected node in Domain-Net corresponds to a putative Pfam-B domain (Pfam ID: PB005666), which connects with six domains including TTSSLRR, NEL, Lipase_GDSL, Sif, Tox_PLDMTX and Pfam-B domain PB006720. With the accumulation of known T3SE data, we speculate these networks will be more complete. By combining with network analysis tools, they can become useful tools for analyzing the functional and evolutionary relationship among T3SEs. For example, even though most domains are specific to animal or plant pathogen T3SEs, there are also some domains such as YopJ domain (Pfam ID: PF03421), which has a serine/threonine acetyltransferase activity and can block host immune signaling by inhibiting kinase phosphorylation (37), shared by both plant pathogen and animal pathogen T3SEs (Figure 3D). This observation suggests that acetylation of host kinase is a prevalent strategy used by pathogens.

## Conclusions

Here, we present BEAN 2.0 as an accurate, practical and convenient bioinformatics platform for T3SE research (Supplementary Figure S7). First, BEAN 2.0 provides a highly accurate predictor. While machine-learning T3SE predictors have been developed in the past several years, we show traditional sequence alignment and domain analysis can substantially improve prediction accuracy if they are integrated with machine-learning predictors in a rational way. Second, BEAN 2.0 integrates with other bioinformatics tools providing a comprehensive analysis platform to timely annotate T3SEs in the three most important aspects: subcellular locations in host cell, disorder regions and functional domains. Finally, BEAN 2.0 stores 1215 verified T3SEs and allows users to explore their relationships through two interactive T3SE-related networks. With the rapid progress of genome sequencing, the validated T3SEs are accumulating at unprecedented rates. There is a higher requirement for systematically summarizing these T3SEs and extracting new knowledge about the evolution of T3SEs. The data resources deposited in BEAN 2.0 represent our first step in the direction.

## Funding

This work was supported by grants from the National Natural Science Foundation of China (31271414 and 31471249).

*Conflict of interest*. None declared.

## Supplementary Data

Supplementary data are available at *Database* Online.
